# Inflammatory Markers as Predictors of Severe Acute Pancreatitis: A Cohort Study

**DOI:** 10.7759/cureus.105607

**Published:** 2026-03-21

**Authors:** Muhammad Amad Afridi, Faryal Sohail, Mahrukh Ali, Afnan Ahmed, Muhammad Umar, Zurnish Rauf, Tosin Ayantoyinbo, Ayesha Israr, Muhammad Ali Sumbal, Atiya Baig

**Affiliations:** 1 General Surgery, York and Scarborough Teaching Hospitals NHS Foundation Trust, York, GBR; 2 internal medicine, Jinnah Sindh Medical University, Karachi, PAK; 3 Internal Medicine, Central Park Medical College, Lahore, PAK; 4 Internal Medicine, Allama Iqbal Medical College, Lahore, PAK; 5 Internal Medicine, Royal Bournemouth Hospital, Bournemouth, GBR; 6 Surgery and Medicine, Jinnah Hospital, Lahore, PAK; 7 Internal Medicine, Obafemi Awolowo College of Health Sciences, Olabisi Onabanjo University, Ago-Iwoye, NGA; 8 Anatomy, Hazrat Bari Imam Sarkar (HBS) Medical and Dental College, Islamabad, PAK; 9 Medicine, Balfour Hospital, Kirkwall, GBR; 10 Internal Medicine, Kulsoom International Hospital, Islamabad, PAK

**Keywords:** acute pancreatitis, c-reactive protein, inflammatory markers, neutrophil-to-lymphocyte ratio, platelet-to-lymphocyte ratio, severe acute pancreatitis

## Abstract

Background: Severe acute pancreatitis (SAP) is a clinical dilemma that is hard to diagnose early and is associated with high morbidity and mortality. There are readily accessible inflammatory markers that could help with timely and effective management and risk stratification.

Purpose: The purpose of the study is to determine the predictive value of neutrophil-to-lymphocyte ratio (NLR), platelet-to-lymphocyte ratio (PLR), and C-reactive protein (CRP) in determining the severity of acute pancreatitis (AP).

Methods: This retrospective multicenter cohort study included 272 adult patients admitted to two tertiary care hospitals in Pakistan (Lahore, n=142; Islamabad, n=130) with confirmed AP between September 2025 and December 2025. The revised Atlanta Criteria were used to classify the disease as severe or non-severe. Admission values were used to study the initial 24-hour values of the NLR, PLR, and CRP. Group comparisons were performed using nonparametric tests, and Spearman correlations were used; univariable logistic regression was employed to assess predictive performance given the high multicollinearity among predictors.

Findings: Patients with SAP had a significantly greater median NLR when compared to those with non-SAP (Mdn 9.4 vs 4.8; U = 1026.50, p < 0.001). The median CRP was also higher among severe ones (Mdn 168mg/L vs 68mg/L; U = 1013.50, p < 0.001). NLR (r = 0.766) and CRP (r = 0.767) showed strong positive correlations with severity. A high predictive accuracy (95.2%) was observed in the study dataset, with elevated odds of severe pancreatitis associated with NLR (OR = 3.78, 95% CI: 2.83-5.06) and CRP (OR = 1.06, 95% CI: 1.05-1.08). PLR had a weak negative association with severity (0.208) and reduced predictive accuracy (74.6%). Male patients (82/155 (52.9%); χ²(1) = 10.840, p = 0.001) and patients aged 60 years and older (65/102 (63.7%); χ²(3) = 10.930, p = 0.012) had higher chances of developing severe disease. Further analyses revealed that these subgroups also exhibited significantly higher median NLR and CRP levels than those of female patients and younger patients (p < 0.05), indicating that demographic risk factors are associated with elevated inflammatory responses.

Conclusion: Elevated NLR and CRP on admission were strongly associated with SAP and demonstrated higher predictive performance than PLR. These findings suggest that simple inflammatory markers may assist early risk stratification, although further prospective validation is required.

## Introduction

Acute pancreatitis (AP) is an acute inflammatory process of the pancreas that alternates between a mild and self-limiting illness with great mortality and multi-organ dysfunction [1]. The occurrence of AP has been on the rise all over the world, making it an enormous burden on the health care systems in terms of intensive care needs and length of stay [2,3]. It is important to screen at-risk patients with severe acute pancreatitis (SAP) early, as timely treatment can improve patient outcomes and reduce complications [4,5].

Inflammatory biomarkers, such as C-reactive protein (CRP), neutrophil-to-lymphocyte ratio (NLR), platelet-to-lymphocyte ratio (PLR), and white blood cell (WBC) count, have been increasingly studied as predictive indices of disease severity in AP [6,7].

These biomarkers are readily available, inexpensive, and reflect the systemic inflammatory response associated with pancreatic injury [8]. Several investigations have suggested that the high levels of these markers are related to the emergence of severe disease, dysfunction of organs, and high morbidity. Still, the findings have been mixed, and generalized predictive thresholds are not firmly defined, likely due to differences in measurement timing, patient populations, and methodological approaches across studies [6,9].

Given the pressing need for effective early predictors of severity, this cohort study assesses the prognostic value of regularly measured inflammatory markers for SAP. The availability of accessible and effective biomarkers can enable clinicians to stratify patients at admission, improve resource allocation, and implement focused management approaches to enhance patient outcomes.

Novelty and rationale

Although many studies examine the role of inflammatory markers in AP, many have been constrained by small sample sizes, single-centre designs, or non-uniform measurement times, leading to inconsistent predictive accuracy. Also, no further rigorous cohort studies have been conducted that simultaneously examine several routine inflammatory biomarkers, such as CRP, NLR, and PLR, to assess their relative predictive value in SAP.

This study addresses these gaps by retrospectively analyzing a multicenter cohort of patients with AP to evaluate the predictive value of commonly available inflammatory biomarkers. The novelty of this retrospective study lies in simultaneously evaluating multiple inflammatory markers in a well-defined cohort and identifying thresholds that may inform future clinical decision-making. The results can enhance early risk stratification, inform prompt interventions, and minimize morbidity and mortality in SAP.

Objectives

The primary objective of this study is to evaluate the relationship between admission inflammatory markers, NLR, PLR, and CRP, and the severity of AP, and to assess their predictive value for identifying patients at risk of severe disease at the time of admission. Secondary objectives include describing the demographic and clinical characteristics of patients and examining their association with disease severity.

## Materials and methods

Study design and setting

The study was a retrospective multicenter cohort conducted at two tertiary care hospitals, one in Lahore and one in Islamabad. Of the 272 included patients, 142 were admitted to the hospital in Lahore and 130 to the hospital in Islamabad. Medical records of patients consecutively admitted with AP between September 2025 and December 2025 were reviewed. The study objectives were to establish a relationship between inflammatory markers at admission and disease severity and to assess the predictive value of these markers for SAP.

Study population

**Figure 1:**
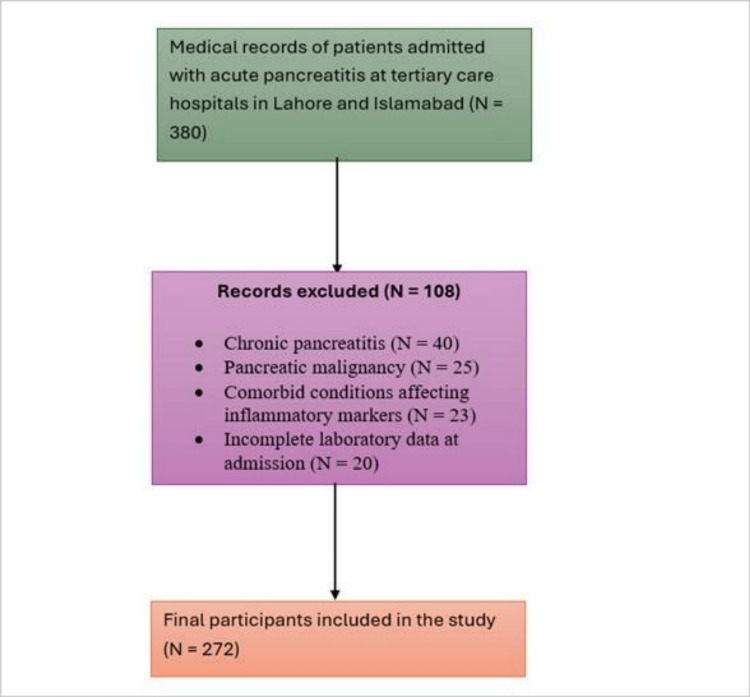
Flowchart Illustrating Participant Selection for the Retrospective Study. Of 380 Records Reviewed, 108 Were Excluded Based on the Eligibility Criteria Shown, Leaving 272 Participants for Final Analysis

A total of 272 participants were included in the sample. Figure 1 illustrates the selection process for the 272 participants included in the study. The study participants were adult patients with a confirmed diagnosis of AP, as determined by clinical presentation (abdominal pain suggesting pancreatitis), laboratory findings (elevated serum amylase and/or lipase), and radiological findings (ultrasound and/or CT scan suggestive of AP). Patients were excluded if they had chronic pancreatitis, pancreatic malignancy, or comorbid conditions that could affect inflammatory markers, such as hematologic disorders or severe systemic infections. These conditions were identified retrospectively through a detailed review of medical records, including clinical notes, past medical history, laboratory findings, and discharge summaries. Additionally, patients with incomplete laboratory data at admission were excluded to ensure reliable assessment of inflammatory indices.

Data collection

Demographic and Clinical Variables

Demographics (age, gender, body mass index (BMI)) were taken as underweight (<18.5 kg/m^2^), normal (18.5 -24.9 kg/m^2^), overweight (25-29.9 kg/m^2^), and obese (≥30 kg/m^2^). Laboratory values were recorded in standard units: neutrophils and lymphocytes ×10^9/L, platelets ×10^9/L, and CRP in mg/L. The lifestyle factors, such as smoking status (current, former, or never) and alcohol use (yes/no), were identified using admission and progress records. The presence of comorbidities, which could affect the disease severity and body inflammation as a whole, e.g., diabetes mellitus, hypertension, and dyslipidemia, was recorded.

Disease severity assessment

The severity of AP was computed according to the Revised Atlanta Classification (2012), which assesses severity based on the presence and duration of organ failure and local or systemic complications [10]. To analyze disease severity, they were divided into two groups: non-SAP (mild and moderately severe pancreatitis) and SAP, defined as persistent organ failure lasting over 48 hours.

Daily progress notes, ICU records, and clinical documentation were evaluated to determine the presence, type, and duration of organ failure, respiratory, renal, and cardiovascular dysfunction. This enabled proper categorization of patients into severe and non-severe patients. Operational definitions were used to classify organ failure: respiratory failure was defined as PaO2/FiO2 <300 mmHg or requirement for mechanical ventilation; renal failure as serum creatinine >1.9 mg/dL or requirement for dialysis; cardiovascular failure as systolic blood pressure <90 mmHg requiring vasopressors [10]. These assessments were verified using ICU records, daily progress notes, and discharge summaries.

Laboratory data and calculation of inflammatory markers

Blood samples were collected via venous puncture into EDTA tubes and analyzed using an automated hematology analyzer. CRP was measured using an immunoturbidimetric method. The first available values within 24 hours of admission were used. Laboratory reference ranges were as follows: neutrophils 2-7×10^9/L, lymphocytes 1-4×10^9/L, platelets 150-450×10^9/L, CRP <5 mg/L. Electronic medical records provided the laboratory values during the initial 24 hours of hospitalization. These consisted of part of the complete blood count (CBC), which includes neutrophils, lymphocytes, and platelets, and CRP. The NLR index, computed as the number of neutrophils divided by the number of lymphocytes, and the PLR index, calculated as the number of platelets divided by the number of lymphocytes, were both used as inflammatory indices. If there were several laboratory values in the first 24 hours, the values from the admission (first) period were used in all calculations to ensure consistency and accurately reflect the patient's inflammatory state during that period.

Outcomes

The main finding of the research was a correlation between the inflammatory markers (NLR, PLR, and CRP) at admission and the severity of AP. The patients were categorized according to the Revised Atlanta Classification, with non-severe (mild or moderately severe) and severe (persistent organ failure >48 hours) as the severity categories.

Statistical analysis

Data analysis was performed using IBM SPSS Statistics for Windows, Version 26 (Released 2018; IBM Corp., Armonk, New York, United States). Continuous variables (NLR, PLR, CRP) were presented as mean ± SD and median (IQR), and categorical variables as frequencies and percentages. Normality of continuous variables was assessed using the Shapiro-Wilk test. Differences between severe and non-severe groups were evaluated using the Mann-Whitney U test. Correlations between inflammatory markers and disease severity were assessed using Spearman's rank correlation. Correlations were calculated using the raw laboratory values of NLR and CRP without transformation or standardization. The dataset was reviewed prior to analysis to ensure that no duplicate or derived variables were used in the correlation calculations. Univariable logistic regression models were used to calculate odds ratios (ORs) with 95% confidence intervals (CIs) for each marker. Multivariable modeling including NLR and CRP simultaneously was not performed because of the extremely high intercorrelation between these variables (ρ = 0.992), which could result in severe multicollinearity and unstable parameter estimates. Therefore, univariable logistic regression models were used to explore the associations between each inflammatory marker and disease severity. Prior to analysis, the dataset was reviewed for potential data entry or calculation errors. Raw laboratory values were used to compute inflammatory markers, and no data transformations were applied. The Spearman correlation between NLR and CRP was rechecked using the original dataset to verify the calculated value. Patients with missing laboratory values were excluded from the analysis. All analyses were conducted using continuous values of inflammatory markers, and no predefined cutoff thresholds were applied. A significance level of p<0.05 was considered statistically significant.

Ethical considerations

The study was approved by the institutional ethics committee of Allama Iqbal Medical College (Ref no: AIMC/IRB/2025/317), and the confidentiality of the data was ensured through de-identification. The study was retrospective, and therefore, no individual patient consent was required.

## Results

As revealed in Table 1, the sample size of the study consisted of 272 individuals divided into the two most prevalent categories of 50-59 (N = 100, 36.8%) and 60 and above (N = 102, 37.5%), and the common subsets of 30-39 (N = 52, 19.1%), and 40-49 (N = 18, 6.6%). The sample consisted of 155 (57.0%) male patients and 117 (43.0%) female patients. Regarding body mass index, 8 (2.9%) were underweight, 68 (25.0%) were normal, 114 (41.9%) were overweight, and 82 (30.1%) were obese. Regarding lifestyle variables, 106 (39.0%) respondents are current smokers, 49 (18.0%) are former smokers, and 117 (43.0%) are non-smokers. One-hundred and sixty-nine (62.1%) respondents consume alcohol continuously, and 103 (37.9%) do not. A few of the comorbidities included diabetes mellitus (N = 139, 51.1%), hypertension (N = 140, 51.5%), and dyslipidemia (N = 155, 57.0%). The severity of AP was non-severe in 151 patients (55.5%) and severe in 121 patients (44.5%).

**Table 1:** Demographic and Clinical Characteristics (N = 272) Data are presented as the number of participants (N) and percentage (%).

Characteristic	n	%
Age (years)		
30–39	52	19.1
40–49	18	6.6
50–59	100	36.8
60 and above	102	37.5
Gender		
Male	155	57.0
Female	117	43.0
Body Mass Index (kg/m²)		
Underweight (<18.5)	8	2.9
Regular (18.5–24.9)	68	25.0
Overweight (25–29.9)	114	41.9
Obese (≥30)	82	30.1
Smoking Status		
Yes	106	39.0
No	117	43.0
Former	49	18.0
Alcohol Use		
Yes	169	62.1
No	103	37.9
Comorbidities		
Diabetes Mellitus	139	51.1
Hypertension	140	51.5
Dyslipidemia	155	57.0
Acute Pancreatitis Severity		
Non-severe	151	55.5
Severe	121	44.5

Table 2 presents the distribution of inflammatory markers according to severity status. Patients with SAP demonstrated higher NLR and CRP values compared with those with non-severe disease in both mean and median measures, indicating a greater inflammatory response in the severe group. In contrast, PLR values were slightly higher in the non-severe group than in the severe group. This pattern suggests that NLR and CRP may be more strongly associated with disease severity, whereas PLR did not show a similar trend in this sample.

**Table 2:** Inflammatory Markers by Severity Status Data are presented as mean (M) ± standard deviation (SD), median (Mdn) with interquartile range (IQR), and range. NLR: neutrophil-to-lymphocyte ratio; CRP: C-reactive protein; PLR: platelet-to-lymphocyte ratio. Descriptive statistics summarizing inflammatory markers across severity groups.

Marker	Severe (n = 121)	Non-severe (n = 151)
NLR		
Mean (SD)	9.600 (1.620)	5.150 (1.250)
Median (IQR)	9.400 (2.700)	4.800 (1.700)
Range	4.1–11.0	3.8–11.0
CRP (mg/L)		
Mean (SD)	157.780 (18.500)	74.190 (28.110)
Median (IQR)	168.000 (40.000)	68.000 (43.000)
Range	48–195	45–195
PLR		
Mean (SD)	232.980 (44.500)	256.250 (26.660)
Median (IQR)	240.000 (85.000)	264.000 (33.000)
Range	144–327	144–327

Table 3 indicates that none of the inflammatory markers were normally distributed across severity groups. The statistics for NLR, CRP, and PLR, both in severe and non-severe patients, were statistically significant (all p < 0.001), indicating that the values are not normally distributed. The findings suggest that group comparisons are done using nonparametric statistics, and central tendency is reported by the use of median and interquartile range.

**Table 3:** Normality Tests for Inflammatory Markers by Severity Status W: Shapiro–Wilk test statistic; p-value < 0.05 indicates deviation from normality. None of the inflammatory markers were normally distributed in either severity group. NLR: neutrophil-to-lymphocyte ratio; CRP: C-reactive protein; PLR: platelet-to-lymphocyte ratio.

Variable	Severity	Shapiro-Wilk Test W (p-value)	Conclusion
NLR	Severe	0.879 (<0.001)	Not normally distributed
-	Non-severe	0.817 (<0.001)	Not normally distributed
CRP (mg/L)	Severe	0.859 (<0.001)	Not normally distributed
-	Non-severe	0.846 (<0.001)	Not normally distributed
PLR	Severe	0.924 (<0.001)	Not normally distributed
-	Non-severe	0.748 (<0.001)	Not normally distributed

Table 4 presents the Spearman rank correlations among inflammatory markers and disease severity. Both NLR and CRP showed strong positive correlations with disease severity (ρ = 0.766 and ρ = 0.767, respectively; p < 0.01), indicating that higher levels of these markers were associated with more SAP. A strong positive correlation was also observed between NLR and CRP (ρ = 0.812, p < 0.01), suggesting that these biomarkers are closely related indicators of inflammatory activity. In contrast, PLR demonstrated a weak but statistically significant negative correlation with disease severity (ρ = −0.208, p < 0.01), indicating that higher disease severity was associated with slightly lower PLR values in this sample.

**Table 4:** Spearman Rank Correlations Among Inflammatory Markers and Severity (N = 272) **p < 0.01 (two-tailed). Spearman rank correlation was used to assess associations among variables. All correlations are statistically significant at the 0.01 level. NLR: neutrophil-to-lymphocyte ratio; CRP: C-reactive protein; PLR: platelet-to-lymphocyte ratio.

Variable	NLR	CRP (mg/L)	PLR	Severity
NLR	1	0.812**	−0.169**	0.766**
CRP (mg/L)	0.812**	1	−0.211**	0.767**
PLR	−0.169**	−0.211**	1	−0.208**
Severity	0.766**	0.767**	−0.208**	1

Table 5 demonstrates statistically significant inflammatory marker differences between severe and non-severe acute pancreatitis. The severe group showed a significantly higher NLR (U = 1026.50, z = -12.61, p < 0.001) and CRP (U = 1013.50, z = -12.62, p < 0.001), in which the effect sizes were also very large (r = 0.760 and r = 0.770, respectively). There was also a difference between groups in PLR (U = 6947.50, z = -3.42, p < 0.001); nevertheless, the magnitude of the effect was low (r = 0.210), which suggests that it is less associated with the severity of the disease than NLR and CRP.

**Table 5:** Mann-Whitney U Tests: Inflammatory Markers and Severity The Mann–Whitney U test was used to compare inflammatory markers between the severe and non-severe groups because the distributions were non-normal. Effect size (r) indicates the magnitude of group differences.

Variable	U Statistic	z	p	Effect Size (r)	Decision
NLR	1026.50	−12.61	<0.001	0.760	Reject H₀
CRP (mg/L)	1013.50	−12.62	<0.001	0.770	Reject H₀
PLR	6947.50	−3.42	<0.001	0.210	Reject H₀

Table 6 shows the univariable logistic regression results assessing the inflammatory markers at admission as predictors of SAP. An increased NLR (OR = 3.778, 95% CI: 2.821-5.059; p < 0.001) and CRP (OR = 1.063, 95% CI: 1.049-1.077; p < 0.001) were both found to give a greater likelihood of severe disease and demonstrated high-performance models (Nagelkerke R 2 > 0.76; accuracy = 95.2%). On the contrary, PLR had a weaker relationship with severity (OR = 0.987, 95% CI = 0.981-0.993; p = 0.001) and a reduced explanatory capacity (R² = 0.094). The statistical significance of all predictors was not less than the significance level corrected with Bonferroni (p < 0.017).

**Table 6:** Univariable Binary Logistic Regression: Inflammatory Markers Predicting Severe Acute Pancreatitis (N = 272) All three univariable logistic regression models were statistically significant (p < .001). NLR and CRP demonstrated excellent predictive power with Nagelkerke R² > .76 and classification accuracy of 95.2%, while PLR showed weak predictive ability with R² = .094 and accuracy of 74.6%. All models showed no evidence of perfect separation with reasonable standard errors. Odds ratios represent the multiplicative change in odds of severe pancreatitis for each unit increase in the predictor variable. Bonferroni correction applied (α = .05/3 = .017); all models exceeded this threshold.

Predictor	B	SE	Wald	p	Exp(B)	95% CI for Exp(B)	−2 LL	R²	Accuracy	Sensitivity	Specificity
NLR	1.329	0.149	79.578	<0.001	3.778	2.821–5.059	145.342	0.761	95.2%	94.2%	96.0%
CRP (mg/L)	0.061	0.007	75.939	<0.001	1.063	1.049–1.077	141.852	0.768	95.2%	94.2%	96.0%
PLR	−0.013	0.003	17.878	<0.001	0.987	0.981–0.993	353.949	0.094	74.6%	49.6%	94.7%

Table 7 presents the multivariable logistic regression analysis evaluating the association between NLR and SAP after adjusting for demographic and clinical confounders. Higher NLR remained an independent predictor of severe disease (aOR = 2.943, 95% CI: 1.547-5.598; p < 0.001), indicating that increased NLR levels were associated with significantly greater odds of SAP. Among the covariates, age ≥60 years (aOR = 2.181, 95% CI: 1.330-3.576; p = 0.002) and male sex (aOR = 1.748, 95% CI: 1.150-2.658; p = 0.009) were also significantly associated with higher odds of severe disease. Obesity (p = 0.053) and alcohol use (p = 0.060) showed borderline associations with severity, whereas other factors including age 40-59 years, overweight BMI, diabetes mellitus, hypertension, and current smoking were not statistically significant predictors in the model. The model demonstrated good fit according to the Hosmer-Lemeshow test (χ²(8) = 6.412, p = 0.601).

**Table 7:** Multivariable Logistic Regression Model 1: NLR Adjusted for Confounders (N = 272) aOR: adjusted odds ratio; CI: confidence interval; SE: standard error; NLR: neutrophil-to-lymphocyte ratio; BMI: body mass index. Reference categories: age <40 years, female sex, and normal BMI. p-values are derived from Wald tests.

Variable	aOR	95% CI	SE	Wald	p
NLR	2.943	1.547–5.598	0.328	10.828	<0.001
Age (≥60 vs. <40 years)	2.181	1.330–3.576	0.252	9.550	0.002
Age (50–59 vs. <40 years)	1.487	0.895–2.472	0.259	2.341	0.126
Age (40–49 vs. <40 years)	1.204	0.488–2.970	0.461	0.162	0.687
Male sex	1.748	1.150–2.658	0.214	6.823	0.009
BMI: Obese vs. Normal	1.651	0.994–2.744	0.259	3.744	0.053
BMI: Overweight vs. Normal	1.382	0.866–2.206	0.239	1.840	0.175
Diabetes mellitus	1.423	0.918–2.207	0.224	2.484	0.115
Hypertension	1.196	0.752–1.903	0.237	0.571	0.450
Alcohol use (Yes vs. No)	1.634	0.980–2.726	0.261	3.537	0.060
Current smoking	1.289	0.809–2.055	0.238	1.138	0.286

Table 8 presents the multivariable logistic regression model evaluating the association between CRP and SAP after adjusting for demographic and clinical confounders. Higher CRP levels were independently associated with increased odds of severe disease (aOR = 1.048, 95% CI: 1.019-1.078; p < 0.001), indicating that each unit increase in CRP was linked to a higher likelihood of SAP. Among the covariates, age ≥60 years (aOR = 2.096, 95% CI: 1.286-3.417; p = 0.003) and male sex (aOR = 1.682, 95% CI: 1.106-2.557; p = 0.015) were also significantly associated with increased odds of severe disease. Obesity (p = 0.074) and alcohol use (p = 0.082) showed borderline associations, whereas other variables including age 40-59 years, overweight BMI, diabetes mellitus, hypertension, and current smoking were not statistically significant predictors in the model. The model demonstrated good fit according to the Hosmer-Lemeshow test (χ²(8) = 7.180, p = 0.518).

**Table 8:** Multivariable Logistic Regression Model 2: CRP Adjusted for Confounders (N = 272) aOR: adjusted odds ratio; CI: confidence interval; SE: standard error; CRP: C-reactive protein; BMI: body mass index. Reference categories: age <40 years, female sex, and normal BMI. p-values are derived from Wald tests.

Variable	aOR	95% CI	SE	Wald	p
CRP (mg/L)	1.048	1.019–1.078	0.014	10.828	<0.001
Age (≥60 vs. <40 years)	2.096	1.286–3.417	0.249	8.807	0.003
Age (50–59 vs. <40 years)	1.421	0.844–2.392	0.266	1.749	0.186
Age (40–49 vs. <40 years)	1.162	0.453–2.984	0.481	0.097	0.755
Male sex	1.682	1.106–2.557	0.214	5.916	0.015
BMI: Obese vs. Normal	1.588	0.956–2.638	0.259	3.192	0.074
BMI: Overweight vs. Normal	1.320	0.827–2.108	0.239	1.352	0.245
Diabetes mellitus	1.389	0.891–2.166	0.227	2.103	0.147
Hypertension	1.147	0.718–1.832	0.239	0.329	0.566
Alcohol use (Yes vs. No)	1.574	0.944–2.624	0.261	3.025	0.082
Current smoking	1.251	0.779–2.009	0.242	0.859	0.354

Table 9 presents the receiver operating characteristic (ROC) curve analysis evaluating the diagnostic performance of inflammatory markers in predicting SAP. Both NLR and CRP demonstrated excellent discriminative ability, with AUC values of 0.944 (95% CI: 0.914-0.974) and 0.945 (95% CI: 0.915-0.974), respectively (p < 0.001 for both). The optimal cutoff value for NLR was ≥6.80, yielding a sensitivity of 92.6% and specificity of 94.7%, while CRP at a cutoff ≥115.0 mg/L showed a sensitivity of 91.7% and specificity of 95.4%. In contrast, PLR demonstrated considerably lower predictive performance with an AUC of 0.620 (95% CI: 0.552-0.687), sensitivity of 52.1%, and specificity of 68.9%, indicating limited discriminative ability. Overall, these findings suggest that NLR and CRP are strong predictors of SAP, whereas PLR has a comparatively weaker predictive value.

**Table 9:** ROC Curve Analysis: Inflammatory Markers Predicting Severe Acute Pancreatitis (N = 272) ROC: receiver operating characteristic; AUC: area under the curve; CI: confidence interval; NLR: neutrophil-to-lymphocyte ratio; CRP: C-reactive protein; PLR: platelet-to-lymphocyte ratio; PPV: positive predictive value; NPV: negative predictive value.

Biomarker	AUC	95% CI	Cutoff	Sensitivity	Specificity	PPV	NPV	p
NLR	0.944	0.914–0.974	≥6.80	92.6%	94.7%	93.3%	94.0%	<0.001
CRP (mg/L)	0.945	0.915–0.974	≥115.0	91.7%	95.4%	94.1%	93.4%	<0.001
PLR	0.620	0.552–0.687	≤220.0	52.1%	68.9%	56.2%	65.4%	<0.001

Table 10 shows that the relationship between gender and AP severity is statistically significant (χ² (1) = 10.840, p = 0.001). There were 82 (67.8%) male patients with severe disease, and 73 (48.3%) with non-severe disease. On the other hand, 78 (51.7%) female patients did not severe disease. These findings indicate that the occurrence of SAP is higher among male patients.

**Table 10:** Gender Distribution by Acute Pancreatitis Severity The chi-square (χ²) test was used to assess the association between gender and disease severity. χ²(1) = 10.840, p = 0.001.

Gender	Severe (n = 121)	Non-severe (n = 151)	Total (N = 272)	p-value
Male	82 (67.8)	73 (48.3)	155 (57.0)	.001
Female	39 (32.2)	78 (51.7)	117 (43.0)	-

Table 11 shows that the age group was significantly correlated with the severity of AP (χ²(3) = 10.930, p = 0.012). The age distribution of severe disease was 12 of 52 (23.1%) in the age range of 30 to 39, 6 of 18 (33.3%) in the 40 65 to 49 age range, 38 of 100 (38.0%) in the 50-59 age range, and 65 of 102 (63.7%) in the age range 60 and over. These findings suggest that elderly patients have a higher risk of getting SAP.

**Table 11:** Age Distribution by Acute Pancreatitis Severity The chi-square (χ²) test was used to assess the association between age group and disease severity. χ²(3) = 10.930, p = 0.012.

Age Group (Years)	Severe (n = 121)	Non-severe (n = 151)	Total (N = 272)	p-value
30–39	12 (9.9)	40 (26.5)	52 (19.1)	.012
40–49	6 (5.0)	12 (7.9)	18 (6.6)	-
50–59	38 (31.4)	62 (41.1)	100 (36.8)	-
60 and above	65 (53.7)	37 (24.5)	102 (37.5)	-

## Discussion

This cohort study evaluated the predictive value of routinely collected inflammatory markers, NLR, CRP, and PLR, to give prognostic data regarding the severity of AP by the Revised Atlanta Classification. In our study, patients with SAP had higher NLR values than those with non-severe cases, indicating greater systemic inflammation. This finding is in line with the recent observations that indicate that greater NLR implies more severe AP, where NLR can be utilized as a marker of the severity of the disease [11]. We found that patients with SAP had higher CRP levels than those with non-SAP, which indicated an elevated level of systemic inflammation. This observation was supported by a survey done previously, where it is said that CRP was a highly sensitive and specific predictor of SAP [12]. PLR did not increase according to the severity of the disease and was slightly lower in SAP as compared to non-severe cases in our study. However, a recent systematic review and meta-analysis suggested that the levels of PLR differed significantly in severe AP, which points to heterogeneity between populations and study designs [13]. This divergence may reflect regional population characteristics, such as variations in baseline hematologic indices, differences in alcohol consumption or comorbidity prevalence, and the distribution of disease severity within the cohort, all of which could influence PLR values.

Our study demonstrated a significant positive correlation between NLR and CRP, indicating that the two were highly correlated in AP. Notably, the observed correlation was extremely high (ρ = 0.812), indicating near collinearity. This may reflect the shared inflammatory dynamics in SAP, where both markers rise in parallel, or could partially result from the timing of laboratory measurements at admission. That is consistent with previous studies, which have found a positive correlation between these markers in AP despite reporting moderate correlations [14]. Such an extremely high correlation between distinct biomarkers is uncommon and may reflect parallel increases in systemic inflammatory responses in SAP, particularly because both markers were measured at admission during the early inflammatory phase. However, this strong correlation also suggests potential collinearity and should therefore be interpreted with caution. Due to the retrospective design and use of univariable regression, we cannot conclude that NLR and CRP are independent predictors of severity, as these associations may be influenced by age, gender, BMI, or comorbidities. In our cohort, PLR showed weak negative correlations with NLR, CRP, and disease severity, suggesting a slight positive relationship between the two. This is contrary to other earlier studies and meta-analyses, which have all reported a high PLR in SAP, being positively correlated with severity [13]. We observe that NLR and CRP are strongly positively correlated with AP severity, which is in line with other studies that found a positive correlation between NLR and CRP and the severity of AP [15]. Conversely, PLR was weakly negatively correlated with seriousness in our cohort, contrary to prior studies, which generally report higher PLR in severe cases, suggesting that this was cohort-specific [15].

NLR and CRP were strongly associated with the severity of AP in our cohort, with group comparisons and univariable logistic regression models demonstrating high predictive accuracy and large effect sizes. These findings are supported by previous studies, including a large retrospective analysis demonstrating high sensitivity and specificity of NLR in severe AP [16] and a meta-analysis of 41 studies showing that CRP is a strong predictor of severe disease [6]. However, these markers were not directly compared with established severity scoring systems such as BISAP, APACHE II, or the Ranson score, and therefore, caution should be exercised when translating these statistical associations into clinical decision-making. Conversely, PLR was a weak predictor and minimally reduced the risk of severe disease, unlike earlier studies that found high PLR to indicate poor prognosis, suggesting that its prognostic performance may have been cohort-dependent [17].

Our study found that NLR is an independent predictor of SAP. This aligns with previous research showing that elevated NLR is significantly associated with severe disease and serves as a prognostic marker, both in large retrospective cohorts and early admission assessments. These findings support the clinical utility of NLR for early risk stratification [16,18]. The association between elevated NLR and SAP may reflect the systemic inflammatory response, where neutrophilia indicates heightened innate immune activation and lymphopenia reflects relative immunosuppression, both of which correlate with more severe disease.

In our study, CRP was an independent predictor of SAP in multivariable logistic regression, with each 1 mg/L increase in CRP associated with a 4.8% higher odds of severe disease. This finding aligns with previous studies reporting that CRP independently predicts SAP in multivariate analyses and serves as a reliable biomarker for early risk stratification [6]. Together, these results support the clinical utility of CRP for identifying patients at risk of severe disease.

In our research, more male patients had SAP than female patients, and this is in accordance with the existing large-scale evidence that indicates greater severity and complications in male patients with this illness. In contrast, female patients were less exposed to adverse effects [19]. In line with prior systematic reviews, the severity of AP in our cohort treatment rose with age, and most of the SAP cases were found in patients aged ≥60 years [20]. This confirms that old age is a risk factor for severe disease on its own, as indicated by various scoring systems.

Altogether, the findings show that NLR and CRP were significantly associated with severity and may serve as early indicators; however, these findings are preliminary and require confirmation in prospective, multivariable, and externally validated studies. Future prospective multicenter studies incorporating multivariable modeling and ROC-based threshold determination are warranted.

Limitations

There are several limitations to this study. To begin with, its retrospective design limits the ability to control for confounding factors, and the results obtained are subject to the completeness of the medical records. Second, the study was conducted in tertiary care hospitals in Lahore and Islamabad, which may limit its generalizability to other healthcare systems or community hospital settings. Third, inflammatory markers were measured only from admission values within the first 24 hours; serial measurements were not performed, and dynamic trends in CRP or hematologic indices may better predict disease progression. Fourth, no comparison between the existing severity scoring systems (including BISAP, APACHE II, and the Ranson score) was performed in the study, which limited the ability to compare the performance of biomarkers with the existing valid clinical instruments. Fifth, although major comorbidities were excluded, residual confounding from metabolic disorders, infections, medications, or other unknown inflammatory processes may remain. Sixth, univariable logistic regression models were used without adjustment for age, gender, BMI, alcohol use, or comorbidities, all of which were independently associated with severity in our cohort; as a result, the reported effect estimates for NLR and CRP may be partially confounded. Seventh, no predefined clinical cutoff thresholds for NLR or CRP were applied, limiting direct clinical applicability. Finally, the reported predictive accuracy reflects internal, in-sample performance only; no external validation or cross-validation was performed, and therefore, the predictive strength may be overestimated in other populations. Despite these limitations, the study provides preliminary evidence supporting the prognostic value of readily available inflammatory markers, which should be confirmed in prospective, multicenter studies with multivariable adjustment and validation.

Future directions

Further studies are needed to confirm these results through future multicenter study designs with larger, more heterogeneous populations. The benefit of serial monitoring of NLR, CRP, and PLR at several time points (e.g., admission, 24 hours, 48 hours, 72 hours) may be to enhance the interpretation of the trajectory of inflammatory responses and their relationship with severe outcomes.

Future research should also evaluate the predictive ability of NLR and CRP relative to conventional clinical scoring systems and investigate whether combining biomarkers with clinical variables (age, comorbidity, indicators of organ dysfunction) can improve risk prediction. Establishing a simplified admission-based risk model using laboratory and demographic predictors can facilitate rapid triage/decision-making during emergencies and inpatient care. Lastly, further studies are required to clarify the inconsistent role of PLR across populations and to determine whether PLR is more useful in specific subgroups (e.g., etiological groups such as gallstone pancreatitis versus alcohol-related pancreatitis).

## Conclusions

This retrospective cohort study found that admission NLR and CRP were significantly associated with SAP and showed potential as early indicators. However, these findings are preliminary and require confirmation in prospective, multivariable, and externally validated studies before routine clinical application. In contrast, PLR performed poorly in predicting SAP. Severe disease was also strongly associated with older age and male gender. The study shows that NLR and CRP are useful clinical tools for rapidity, accessibility, and cost-effectiveness in identifying initial risk in AP. Routine assessment of inflammatory indices could help identify at-risk patients, expedite care, improve resource allocation, and possibly minimize complications and mortality in SAP.
